# Comparative cardiovascular outcomes of aripiprazole vs. risperidone in patients with type 2 diabetes and schizophrenia: a retrospective cohort study

**DOI:** 10.3389/fphar.2025.1617534

**Published:** 2025-07-28

**Authors:** Yi-Ting Yeh, Shih-Chang Lo, Chien-Ning Huang, Yi-Sun Yang, Pei-Lun Liao, Edy Kornelius

**Affiliations:** ^1^School of Medicine, Chung Shan Medical University, Taichung, Taiwan; ^2^Department of Internal Medicine, Division of Endocrinology and Metabolism, Chung Shan Medical University Hospital, Taichung, Taiwan; ^3^Institute of Medicine, Chung Shan Medical University, Taichung, Taiwan; ^4^Department of Medical Research, Chung Shan Medical University Hospital, Taichung, Taiwan

**Keywords:** aripiprazole, risperidone, MACE, schizophrenia, diabetes

## Abstract

**Background:**

Individuals with schizophrenia have substantially elevated cardiovascular morbidity and mortality, a disparity further exacerbated by coexisting type 2 diabetes mellitus (T2DM). The impact of specific antipsychotics on long-term cardiovascular outcomes in patients with T2DM remains unclear. We aimed to compare major adverse cardiovascular events (MACEs) in patients with co-occurring T2DM and schizophrenia treated with aripiprazole versus risperidone.

**Methods:**

We conducted a multi-center retrospective cohort study within the TriNetX US Collaborative Network (2014–2024). Adults (≥18 years) with diagnoses of T2DM and schizophrenia who were new users of aripiprazole or risperidone were identified. Aripiprazole and risperidone cohorts were propensity score–matched 1:1 (n = 5,691 each) on demographics (age, sex, race/ethnicity, body mass index), healthcare utilization, socioeconomic and lifestyle factors, comorbidities, and baseline medications. The primary outcome was time to first major adverse cardiovascular events, defined as a composite of myocardial infarction, ischemic or hemorrhagic stroke, heart failure, ventricular arrhythmia, sudden cardiac death, or all-cause mortality. Kaplan-Meier estimation and Cox proportional hazards models were used to compare outcomes over up to 10 years of follow-up. Subgroup analyses by sex, age, and race and a time-stratified analysis (≤1 year vs. >1 year follow-up) were performed.

**Results:**

After matching, baseline characteristics were well balanced (mean age 51.1 years, 45% female, median HbA1c ∼7.3% in both groups). Aripiprazole was associated with a significantly elevated hazard of MACE compared to risperidone (hazard ratio [HR] 1.10, 95% confidence interval 1.02–1.18). This risk difference emerged primarily beyond the first year of treatment. The excess risk with aripiprazole was driven largely by higher rates of heart failure and ventricular arrhythmias, whereas risks of myocardial infarction and ischemic stroke were similar between groups. No significant heterogeneity in the treatment effect was observed across sex, age, or racial subgroups.

**Conclusion:**

In this large real-world cohort of patients with T2DM and schizophrenia, aripiprazole use was associated with a modest but significant increase in the risk of MACEs compared to risperidone. Clinicians should remain vigilant about cardiovascular risk management in this population regardless of antipsychotic choice. Further research is needed to elucidate mechanisms and to confirm these observations in prospective studies.

## Introduction

Cardiovascular disease (CVD) is the leading cause of premature mortality in people with schizophrenia, accounting for roughly 50% of all deaths in this population (Vohra, 2020). Individuals with schizophrenia experience a reduction in life expectancy of approximately 15–20 years compared to the general population (Laursen, 2011). This excess mortality is primarily attributable to cardiometabolic conditions, influenced by both the pathophysiology of schizophrenia and the adverse effects of its treatments. Schizophrenia is associated to unhealthy lifestyle behaviors and a high prevalence of traditional cardiovascular risk factors, such as obesity, smoking, dyslipidemia, and type 2 diabetes mellitus (T2DM). These risk factors are often under-recognized and inadequately managed in psychiatric settings (Correll et al., 2022). Notably, the odds of developing T2DM are about twofold higher in people with schizophrenia relative to the general population (Dong et al., 2024). The coexistence of schizophrenia and T2DM confers additive risk, patients with both conditions represent a particularly vulnerable group for CVD complications.

Antipsychotic medications, the cornerstone of schizophrenia treatment, can themselves exacerbate cardiometabolic risk (Pasternak et al., 2014). Many second-generation antipsychotics (SGAs) induce weight gain, hyperglycemia, and dyslipidemia (De Hert et al., 2012; American Diabetes Association, 2004; Lambert et al., 2006; Scigliano and Ronchetti, 2013). However, SGAs are heterogeneous in their metabolic effects: agents such as clozapine and olanzapine carry the highest metabolic liability, while others like risperidone have moderate risk, and aripiprazole and ziprasidone are associated with a smaller increase in metabolic risk (De Hert et al., 2012). Aripiprazole, a dopamine D_2_ partial agonist, is often considered a metabolically “weight-sparing” antipsychotic. For example, meta-analyses have found that aripiprazole treatment tends to cause less weight gain and adverse metabolic change than some D_2_-antagonist antipsychotics (including risperidone) (Kim et al., 2021; Swainston Harr et al., 2004). This has led to the expectation that aripiprazole might be a safer choice in patients at high cardiometabolic risk, such as those with T2DM. At the same time, it is recognized that all antipsychotics can negatively affect cardiometabolic health to some degree and that untreated psychosis itself is detrimental (Scigliano and Ronchetti, 2013). Indeed, consistent antipsychotic use has been associated with improved overall survival in schizophrenia despite potential side effects, presumably by reducing psychiatric relapse, suicide, and behavioral harms (Correll et al., 2022).

Comparative data on the long-term cardiovascular outcomes of specific antipsychotics in high-risk populations remain limited (Pasternak et al., 2014). Prior studies in broader patient populations have yielded mixed results. In one retrospective claims-based analysis, aripiprazole users had lower incidence of certain cardiovascular events than users of several other SGAs; notably, risperidone was associated with higher risks of stroke, heart failure, and any cardiovascular event relative to aripiprazole (Citrome et al., 2013). Clinicians managing patients with schizophrenia and T2DM must balance glycemic and cardiovascular considerations when selecting antipsychotic therapy, yet direct evidence to inform this decision has been lacking.

We therefore conducted a retrospective cohort study, to compare cardiovascular outcomes between two commonly used antipsychotics, aripiprazole and risperidone in patients with coexisting T2DM and schizophrenia. We focused on the occurrence of major adverse cardiovascular events (MACEs), a composite outcome encompassing cardiovascular mortality and major morbidity. We hypothesized that risperidone, with relatively greater metabolic side effects, might be associated with a higher rate of MACEs compared to aripiprazole. Alternatively, given recent observations and the complex pharmacology of these drugs, it was equally possible that no significant difference or even the opposite trend could be observed. The goal of this study was to provide real-world evidence to guide antipsychotic selection and risk mitigation in this dual-diagnosis population.

## Materials and methods

### Study design and data source

This was a retrospective cohort study utilizing the TriNetX^®^ US Collaborative Network, a federated health research platform that aggregates de-identified electronic health record (EHR) data from healthcare organizations across the United States (Ludwig et al., 2025). The network includes data on over 150 million patients, predominantly from large academic medical centers, and provides real-time access to clinical variables including diagnoses, procedures, medications, laboratory results, and vital status. The study period spanned 1 January 2014 through 31 December 2024. The study protocol was approved by the Institutional Review Board of Chung Shan Medical University Hospital, identified by the reference number CS2-23159.

#### Cohort selection

Patients were eligible if they had documented diagnoses of schizophrenia (International Classification of Diseases codes F20.x) and type 2 diabetes mellitus (ICD codes E11.x) and were initiated on either aripiprazole or risperidone during the study period. The index date was defined as the date of the first prescription or administration of either aripiprazole or risperidone on or after 1 January 2014, following the diagnosis of both schizophrenia and T2DM. We restricted to new users of the antipsychotic to better capture incident exposure and outcomes, which operationalized as no record of the respective drug in the year prior to index. Patients <18 years old at index were excluded. To ensure mutually exclusive exposure groups, we excluded patients who received both aripiprazole and risperidone at any point after the index date. Thus, patients who switched from one antipsychotic to the other during follow-up were not included in the final analysis. Only those who remained on their index medication (either aripiprazole or risperidone) without switching were retained. We excluded individuals with any documented MACE within 1 year before the index date to focus on new-onset events, as well as those who were recorded as deceased prior to the index date.

**FIGURE 1:**
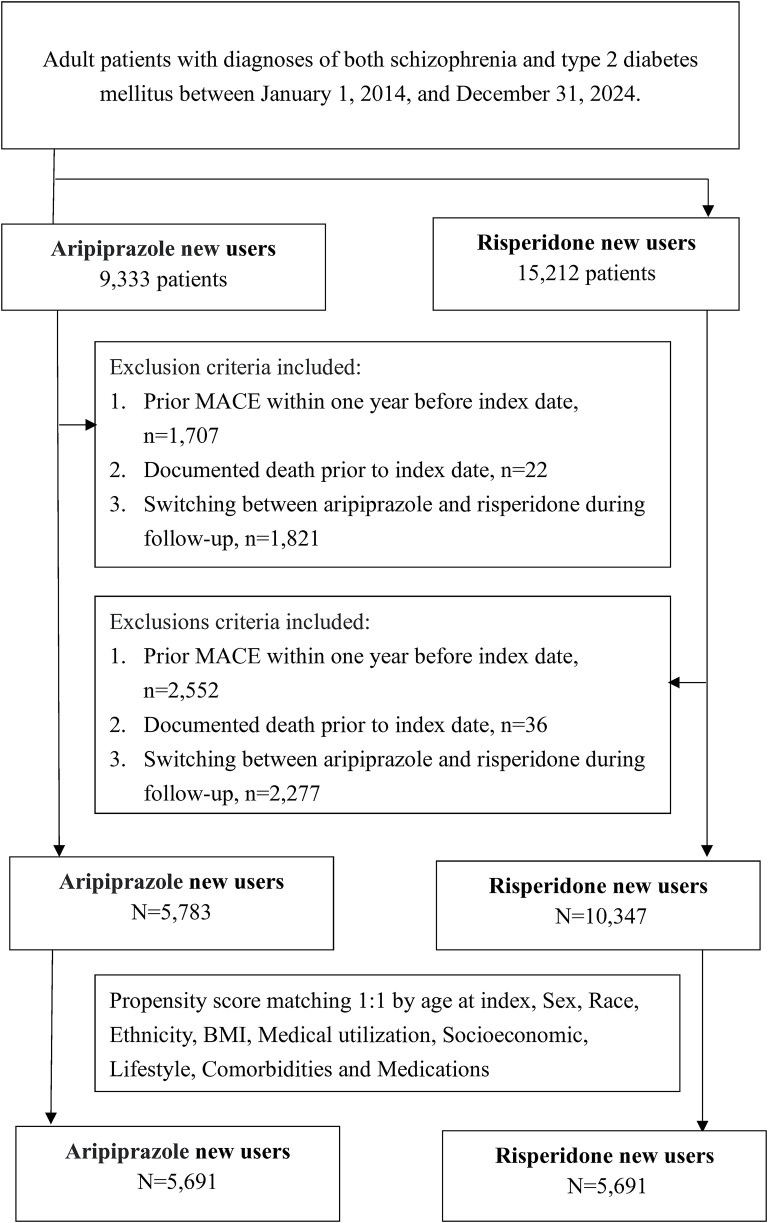
Study flow diagram of cohort selection.

Using these criteria, we identified two cohorts: 9,333 patients initiated on aripiprazole and 15,212 patients initiated on risperidone (Figure 1). After applying exclusions for prior MACE (1,707 and 2,552 patients in the aripiprazole and risperidone groups, respectively), prior death (22 and 36 patients), and cross-use of the alternate antipsychotic (1,821 and 2,277 patients who switched post-index), the final sample for analysis comprised 5,783 aripiprazole users and 10,347 risperidone users before matching. The resulting cohorts were balanced by 1:1 propensity score matching (PSM) for baseline characteristics (demographics, comorbidities, and medications), yielding 5,691 patients in each group for outcome analysis.

We employed an intent-to-treat (ITT) analytical approach for patients who switched to a third antipsychotic (i.e., not the comparator drug). These patients remained classified in their original exposure group throughout the follow-up period, regardless of subsequent medication changes. This approach reflects real-world treatment patterns and supports the evaluation of treatment strategies based on the initial antipsychotic choice. Similarly, patients who discontinued their index antipsychotic without switching to another antipsychotic were not censored at the time of discontinuation and remained in their original exposure group. Follow-up continued for these individuals through the prespecified observation period to maintain analytic consistency with the ITT framework.

### Baseline measures

Baseline was defined as the 1-year period before and including the index date. Patient characteristics extracted from the EHR data included: age at index, sex, race and ethnicity, and body mass index (BMI). We recorded medical utilization metrics (such as the occurrence of any inpatient hospitalization or emergency department visit, and the count of outpatient visits in the prior year) as proxies for healthcare engagement. Socioeconomic and lifestyle factors were captured via diagnosis codes for problems related to education, employment, or housing (ICD-10 Z55–Z59) and for tobacco and alcohol use (Z72.0, F17, F10). We assessed baseline comorbidities including hypertension, dyslipidemia, chronic kidney disease, atrial fibrillation, chronic pulmonary disease (COPD or asthma), liver disease, and other psychiatric diagnoses (major depressive disorder, bipolar disorder, anxiety disorders) using relevant ICD-10 codes (Supplementary Table 1 provides code definitions). Given that all patients had T2DM by inclusion criteria, we further examined indices of diabetes severity: the most recent hemoglobin A1c in the baseline year (mean value and categories <7%, 7%–9%, >9%) and prescriptions for diabetes medications (e.g., metformin, sulfonylureas, DPP-4 inhibitors, GLP-1 agonists, etc.), as well as statin and aspirin use, within the year prior to index.

#### Propensity score matching (PSM)

To address baseline differences between aripiprazole and risperidone users, we performed PSM. A multivariable logistic regression model was used to estimate the propensity to be prescribed aripiprazole (versus risperidone) given baseline covariates. The model included the following covariates: age, sex, race, ethnicity, BMI, indicators of medical utilization, socioeconomic and lifestyle factors, all comorbidities, and use of diabetes and cardiovascular medications. Nearest-neighbor 1:1 matching without replacement was then applied, using a caliper of 0.1 on the propensity score logit. The matching algorithm was executed within the TriNetX analytics platform. Balance between the matched groups was assessed by standardized mean differences (SMDs) for each covariate, with an SMD <0.1 indicating good balance.

### Outcome definition

The primary outcome was the occurrence of a MACE, defined as a composite of: (1) acute myocardial infarction (MI), (2) ischemic stroke, (3) hemorrhagic stroke, (4) heart failure, (5) ventricular arrhythmia or sudden cardiac arrest, or (6) all-cause mortality. These components were defined by diagnosis codes (ICD-10 I21–I22 for MI; I63 and related codes for ischemic stroke; I61–I62 for hemorrhagic stroke; I50 for heart failure; I47.0, I47.2, I49.0, I49.3 for ventricular tachyarrhythmias; I46 or equivalent for sudden cardiac death) or a recorded death in the EHR. The first occurrence of any of these events after the index date was considered an outcome event. Patients were followed from the index date until the first MACE, disenrollment from the health system, or end of the observation period (31 December 2024), whichever came first. Secondary outcomes for descriptive purposes included the individual components of the composite.

### Statistical analysis

We used Kaplan-Meier survival analysis to estimate the cumulative incidence of MACE over time in each matched group, treating non-MACE death as an event in the composite and censoring only at end of follow-up or loss to follow-up. A log-rank test was used to compare unadjusted event-free survival between groups. Cox proportional hazards models were used to estimate the hazard ratio of MACE for aripiprazole versus risperidone in the matched cohort, with robust standard errors to account for the matched design. The proportional hazards assumption was evaluated by visual inspection of log-minus-log plots and time-interaction tests; this revealed a possible divergence in hazards over time. Therefore, we conducted a prespecified time-stratified analysis splitting follow-up into early (day 1–365) and late (day 365 onward) periods, estimating separate Cox models for each interval to see if the treatment effect differed by follow-up time. We also performed subgroup analyses, stratifying the matched cohort by sex (female vs. male), age category (<50, 50–64, ≥65 years), and race (Black, White, and Asian), and calculated hazard ratios within each stratum. Interaction terms between treatment and subgroup indicators were tested in the Cox models to evaluate statistical heterogeneity of the treatment effect.

All analyses were conducted using the TriNetX platform’s built-in analytics and R statistical packages. Two-sided *p* < 0.05 was considered statistically significant. Results are reported as hazard ratios with 95% confidence intervals.

## Results

A total of 11,130 patients (5,783 aripiprazole users and 10,347 risperidone users) met inclusion criteria after exclusions and were eligible for matching (Figure 1). Before matching, there were notable baseline differences between the groups (Table 1). Patients prescribed aripiprazole tended to be slightly younger (mean age 51.0 ± 13.7 years) than those on risperidone (53.5 ± 13.9 years, SMD 0.18). The aripiprazole cohort had a somewhat higher proportion of individuals with obesity (BMI ≥30: 37.3% vs. 29.1%) and with poorly controlled diabetes (baseline HbA1c ≥ 9% in 8.1% vs. 5.5%) compared to the risperidone cohort. They also had higher prevalences of co-occurring psychiatric conditions such as depression (21.0% vs. 15.4%) and anxiety disorders (20.5% vs. 15.3%. Aripiprazole users were more likely to have been on metformin (20.0% vs. 15.9%) and statins (20.0% vs. 16.9%) at baseline, consistent with a greater metabolic burden, and had slightly more outpatient encounters in the prior year (SMD ∼0.10 for outpatient visits) (Table 1). In contrast, risperidone users were more often Black or African American (41.5% vs. 36.7%). The mean follow-up duration was 1,270 days in the aripiprazole group and 1,197 days in the risperidone group. The corresponding median follow-up times were 1,038 and 928 days, respectively. Due to limitations in the dataset, detailed information regarding reasons for censoring, such as medication discontinuation, switching, or loss to follow-up was not available.

**TABLE 1:** Baseline characteristics of patients treated with aripiprazole or risperidone before and after propensity score matching.

	Before PSM^a^			After PSM^a^		
	Aripiprazole	Risperidone	SMD	Aripiprazole	Risperidone	SMD
N	5,783	10,347		5,691	5,691	
Age at Index (Mean±SD)	51.0±13.7	53.5±13.9	0.1841	51.2±13.7	51.0±14.0	0.0088
Sex						
Female	2617(45.3%)	4638(44.8%)	0.0086	2571(45.2%)	2561(45.0%)	0.0035
Male	3003(51.9%)	5457(52.7%)	0.0163	2961(52.0%)	3009(52.9%)	0.0169
Unknown Gender	163(2.8%)	252(2.4%)	0.0240	159(2.8%)	121(2.1%)	0.0431
Ethnicity						
Hispanic or Latino	515(8.9%)	870(8.4%)	0.0177	503(8.8%)	483(8.5%)	0.0125
Not Hispanic or Latino	3995(69.1%)	7312(70.7%)	0.0346	3933(69.1%)	4005(70.4%)	0.0275
Unknown Ethnicity	1273(22.0%)	2165(20.9%)	0.0265	1255(22.1%)	1203(21.1%)	0.0222
Race						
American Indian or Alaska Native	31(0.5%)	34(0.3%)	0.0316	28(0.5%)	29(0.5%)	0.0025
Asian	194(3.4%)	409(4.0%)	0.0319	192(3.4%)	190(3.3%)	0.0020
Black or African American	2123(36.7%)	4297(41.5%)	0.0988	2110(37.1%)	2104(37.0%)	0.0022
Native Hawaiian or Other Pacific Islander	42(0.7%)	84(0.8%)	0.0098	42(0.7%)	34(0.6%)	0.0173
White	2695(46.6%)	4229(40.9%)	0.1157	2635(46.3%)	2667(46.9%)	0.0113
Other Race	193(3.3%)	337(3.3%)	0.0045	184(3.2%)	192(3.4%)	0.0079
Unknown Race	505(8.7%)	957(9.2%)	0.0181	500(8.8%)	475(8.3%)	0.0157
BMI	32.2±8.8	30.6±8.5	0.1773	32.0±8.8	31.8±8.9	0.0210
At most 25 kg/m2	772(13.3%)	1525(14.7%)	0.0400	763(13.4%)	761(13.4%)	0.0010
25-30 kg/m2	985(17.0%)	1816(17.6%)	0.0137	967(17.0%)	965(17.0%)	0.0009
30-35 kg/m2	1002(17.3%)	1473(14.2%)	0.0849	967(17.0%)	936(16.4%)	0.0146
At least 35 kg/m2	1155(20.0%)	1537(14.9%)	0.1353	1096(19.3%)	1082(19.0%)	0.0063
Medical utilization						
Office or Other Outpatient Services	1458(25.2%)	2162(20.9%)	0.1026	1401(24.6%)	1361(23.9%)	0.0164
Hospital Inpatient Services	1142(19.7%)	1761(17.0%)	0.0705	1104(19.4%)	1101(19.3%)	0.0013
Emergency Department Services	2441(42.2%)	4286(41.4%)	0.0160	2381(41.8%)	2348(41.3%)	0.0118
Socioeconomic and psychosocial circumstances	710(12.3%)	1102(10.7%)	0.0511	676(11.9%)	663(11.7%)	0.0071
Problems related to education and literacy	14(0.2%)	21(0.2%)	0.0083	14(0.2%)	14(0.2%)	0.0000
Problems related to employment and unemployment	120(2.1%)	169(1.6%)	0.0327	111(2.0%)	111(2.0%)	0.0000
Occupational exposure to risk factors	10(0.2%)	10(0.1%)	0.0208	10(0.2%)	10(0.2%)	0.0000
Problems related to housing and economic circumstances	453(7.8%)	780(7.5%)	0.0111	440(7.7%)	453(8.0%)	0.0085
Lifestyle						
Tobacco use	273(4.7%)	431(4.2%)	0.0270	266(4.7%)	257(4.5%)	0.0076
Nicotine dependence	1289(22.3%)	2237(21.6%)	0.0162	1262(22.2%)	1284(22.6%)	0.0093
Alcohol related disorders	449(7.8%)	745(7.2%)	0.0214	434(7.6%)	444(7.8%)	0.0066
Comorbidities						
Hypertensive diseases	2445(42.3%)	4133(39.9%)	0.0475	2387(41.9%)	2366(41.6%)	0.0075
Disorders of lipoprotein metabolism and other lipidemias	1579(27.3%)	2570(24.8%)	0.0562	1528(26.8%)	1473(25.9%)	0.0219
Chronic kidney disease (CKD)	379(6.6%)	601(5.8%)	0.0310	368(6.5%)	349(6.1%)	0.0137
Neoplasms	382(6.6%)	630(6.1%)	0.0212	366(6.4%)	369(6.5%)	0.0021
Atrial fibrillation and flutter	111(1.9%)	205(2.0%)	0.0045	110(1.9%)	121(2.1%)	0.0137
chronic obstructive pulmonary disease	445(7.7%)	752(7.3%)	0.0162	429(7.5%)	427(7.5%)	0.0013
Asthma	573(9.9%)	779(7.5%)	0.0844	542(9.5%)	569(10.0%)	0.0160
Diseases of liver	339(5.9%)	380(3.7%)	0.1029	310(5.4%)	310(5.4%)	0.0000
Sleep disorders	816(14.1%)	1049(10.1%)	0.1219	762(13.4%)	765(13.4%)	0.0015
Overweight and obesity	1033(17.9%)	1294(12.5%)	0.1497	970(17.0%)	956(16.8%)	0.0066
Depressive episode	1217(21.0%)	1598(15.4%)	0.1454	1175(20.6%)	1000(17.6%)	0.0783
Bipolar disorder	1028(17.8%)	1531(14.8%)	0.0808	991(17.4%)	972(17.1%)	0.0088
Other anxiety disorders	1187(20.5%)	1588(15.3%)	0.1353	1130(19.9%)	1033(18.2%)	0.0435
Medications						
Biguanides	1157(20.0%)	1649(15.9%)	0.1061	1102(19.4%)	1069(18.8%)	0.0148
Sulfonylureas	289(5.0%)	427(4.1%)	0.0417	277(4.9%)	263(4.6%)	0.0116
Alpha glucosidase inhibitors	10(0.2%)	10(0.1%)	0.0208	10(0.2%)	10(0.2%)	0.0000
Thiazolidinediones	51(0.9%)	55(0.5%)	0.0418	44(0.8%)	43(0.8%)	0.0020
Dipeptidyl peptidase 4 (DPP-4) inhibitors	179(3.1%)	204(2.0%)	0.0716	163(2.9%)	154(2.7%)	0.0096
Glucagon-like peptide-1 (GLP-1) analogues	131(2.3%)	127(1.2%)	0.0793	110(1.9%)	109(1.9%)	0.0013
HMG CoA reductase inhibitors	1155(20.0%)	1750(16.9%)	0.0789	1118(19.6%)	1067(18.7%)	0.0228
Aspirin	640(11.1%)	985(9.5%)	0.0509	622(10.9%)	592(10.4%)	0.0171
Hemoglobin A1c/Hemoglobin.total in Blood (Mean±SD)	7.4±2.7	7.2±2.5	0.0807	7.3±2.6	7.3±2.5	0.0007
At most 7 %	1153(19.9%)	1814(17.5%)	0.0617	1119(19.7%)	1093(19.2%)	0.0115
7-9 %	467(8.1%)	676(6.5%)	0.0593	443(7.8%)	430(7.6%)	0.0086
At least 9 %	471(8.1%)	572(5.5%)	0.1038	438(7.7%)	429(7.5%)	0.0060

^a^ PSM (matching include Age at index, Sex, Ethnicity, Race, BMI, Medical utilization, Socioeconomic, Lifestyle, Comorbidities and Medication). CKD, chronic kidney disease; COPD, chronic obstructive pulmonary disease; SMD, standardized mean difference.

PSM achieved excellent balance on all measured covariates. The matched sample comprised 5,691 patients in each group. In the matched cohort, the mean age was 51.1 years in both groups (±13.8), with 45.2% female in aripiprazole vs. 45.0% in risperidone (SMD <0.01). Racial and ethnic distributions were identical after matching (e.g., 46.3% vs. 46.9% White; 37.1% vs. 37.0% Black; SMD <0.01). Comorbid conditions including hypertension (∼42% each), dyslipidemia (∼26–27%), chronic kidney disease (∼6%), and psychiatric comorbidities (e.g., depression ∼20%, bipolar ∼17% in both) were well balanced (all SMD <0.1). Baseline BMI (mean ∼31.9 in both groups) and glycemic control were also similar post-match, for instance, the proportion with HbA1c ≥ 9% was ∼7.6% in both groups (SMD ∼0.006). The use of glucose-lowering and CV medications did not differ meaningfully after matching (metformin ∼19%, insulin ∼8% in each; statin ∼19% in each). Thus, the matched cohorts were highly comparable in terms of demographic and clinical characteristics at baseline, minimizing confounding in subsequent outcome comparisons.

**FIGURE 2:**
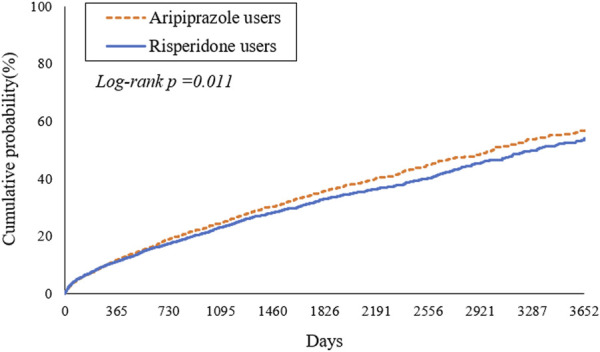
Kaplan-Meier estimates of time to first major adverse cardiovascular events (MACE) in patients with type 2 diabetes mellitus and schizophrenia treated with aripiprazole (dashed line) versus risperidone (solid line).

During the study period, we observed a high cumulative incidence of MACEs in both treatment groups, reflecting the high-risk nature of the study population. By 2 years after antipsychotic initiation, approximately 19.1% of aripiprazole-treated patients and 17.5% of risperidone-treated patients had experienced a MACE. Cumulative incidence curves continued to rise steeply over time (Figure 2). At 6 years, an estimated forty percent of patients in the aripiprazole group had suffered a MACE, compared to ∼36% in the risperidone group. By 10 years of follow-up, the cumulative MACE incidence reached 56.8% in the aripiprazole group versus 54.1% in the risperidone group (Table 2). The Kaplan-Meier curves for the composite outcome (Figure 2) showed that the two groups’ event-free survival was very similar in the early follow-up period, but a gap favoring risperidone began to emerge after approximately 2–3 years on treatment. A log-rank test indicated a statistically significant difference between the survival curves (*p* = 0.011), corresponding to the aripiprazole group having worse CV event-free survival over the long term.

**TABLE 2:** Hazard ratios and cumulative incidence of major adverse cardiovascular events (MACEs) in matched^a^ patients treated with aripiprazole versus risperidone.

	Patients with outcome	Cumulative probability (%) of MACEs since index date					Hazard ratio^b^ (95% CI)
		2 year	4 year	6 year	8 year	10 year	
Patients in cohort, n = 5,691							
MACEs (include Mortality)							
Aripiprazole	1,534	19.1	30.3	40.2	48.7	56.8	**1.10 (1.02–1.18)**
Risperidone	1,367	17.5	28.2	36.6	45.6	54.1	Reference

^a^ Propensity score matching include Age at index, Sex, Ethnicity, Race, BMI, Medical utilization, Socioeconomic, Lifestyle, Comorbidities and Medication.

^b^ Hazard ratio for outcomes among Aripiprazole group compared to Risperidone group subjects (after propensity score matching). 95% CI, 95% confidence interval.

Bold values indicate statistical significance (*p* < 0.05).

In the primary Cox analysis on the matched cohort, aripiprazole was associated with a higher risk of MACEs compared to risperidone. The hazard ratio (HR) was 1.10 (95% confidence interval (CI) 1.02–1.18) in favor of risperidone (with risperidone as reference), indicating a 10% relative increase in hazard with aripiprazole. Notably, the proportional hazard assumption appeared to be violated to a mild degree, the hazard ratio was not constant over time. We therefore examined the hazard within two-time intervals. During the first year following initiation, the incidence of MACE was high in both groups (∼10% in 1 year) but did not significantly differ by treatment: 577 aripiprazole patients vs. 537 risperidone patients had a MACE within 1 year (cumulative incidence ∼10.1% vs. 9.4%), yielding HR 1.04 (0.93–1.17), which was not statistically significant (Table 3). Beyond the first year (from 1 to 10 years post-index), an additional 1,123 aripiprazole patients vs. 999 risperidone patients experienced MACE, and in this later period the HR was 1.10 (1.01–1.20), consistent with a modestly higher long-term risk associated with aripiprazole. In other words, the divergence in outcomes became more pronounced with longer follow-up, suggesting that any potential CV detriment of aripiprazole compared to risperidone manifests over the course of years.

**TABLE 3:** Time-stratified hazard ratios for major adverse cardiovascular events: Day 1–365 and beyond in matched cohorts^a^.

	Follow up 1 day–365 day		Follow up 365 day to anytime	
	Patients in cohort, n = 5,691		Patients in cohort, n = 5,491	
	Patients with outcome	Hazard ratio^b^ (95% CI)	Patients with outcome	Hazard ratio^b^ (95% CI)
MACEs (include Mortality)				
Aripiprazole	577	1.04 (0.93–1.17)	1,123	1.10 (1.01–1.20)
Risperidone	537	Reference	999	Reference
Myocardial infarction				
Aripiprazole	87	0.93 (0.69–1.25)	210	1.15 (0.94–1.41)
Risperidone	90	Reference	173	Reference
Ischemic stroke				
Aripiprazole	119	0.98 (0.76–1.27)	269	1.09 (0.91–1.29)
Risperidone	117	Reference	236	Reference
Hemorrhagic stroke				
Aripiprazole	17	1.63 (0.75–3.57)	45	1.12 (0.73–1.73)
Risperidone	10	Reference	38	Reference
Heart failure				
Aripiprazole	285	1.17 (0.98–1.39)	612	1.28 (1.14–1.45)
Risperidone	236	Reference	463	Reference
Ventricular arrhythmia				
Aripiprazole	86	1.43 (1.03–1.99)	193	1.41 (1.13–1.76)
Risperidone	58	Reference	130	Reference
Sudden cardiac death				
Aripiprazole	137	0.85 (0.67–1.06)	348	0.86 (0.74–1.00)
Risperidone	156	Reference	383	Reference

^a^ Propensity score matching include Age at index, Sex, Ethnicity, Race, BMI, Medical utilization, Socioeconomic, Lifestyle, Comorbidities and Medication.

^b^ Hazard ratio for outcomes among Aripiprazole group compared to Risperidone group subjects (after propensity score matching). 95% CI, 95% confidence interval.

We explored the individual components of the composite outcome to identify which events contributed to the observed difference (Table 4). Heart failure events were notably more frequent in the aripiprazole group. A total of 807 aripiprazole-treated patients were diagnosed with incident heart failure during follow-up, compared to 607 in the risperidone group (after matching). The cumulative incidence of heart failure at 10 years was 33.7% with aripiprazole vs. 27.9% with risperidone. This interpreted to a HR of 1.29 (1.16–1.44) for heart failure (aripiprazole vs. risperidone). The difference in heart failure emerged gradually: the HR in the first year was 1.17 (0.98–1.39) and in years 1+ was 1.28 (1.14–1.45), indicating a significant long-term effect (Table 3). Another driver of the composite outcome was ventricular arrhythmia. We observed 278 patients with ventricular arrhythmia in the aripiprazole group versus 205 in the risperidone group. The 10-year incidence of serious arrhythmias was 15.0% vs. 12.3%, respectively, and aripiprazole was associated with a 29% higher hazard of ventricular arrhythmia (HR 1.29, 95% CI 1.08–1.54). Interestingly, unlike the composite outcome, the excess risk of arrhythmias with aripiprazole was present even in the first year: within 1 year, 86 aripiprazole patients vs. 58 risperidone patients had a ventricular arrhythmia (HR 1.43, 1.03–1.99), and beyond 1 year HR was 1.41 (1.13–1.76) (Table 3). These findings point to a consistent relative increase in arrhythmia events among aripiprazole users.

**TABLE 4:** Hazard ratios and cumulative incidence of individual major adverse cardiovascular events by treatment group.

	Patients with outcome	Cumulative probability (%) of MACEs since index date					Hazard ratio^a^ (95% CI)
		2 year	4 year	6 year	8 year	10 year	
Patients in cohort, n = 5,691							
Myocardial infarction							
Aripiprazole	296	3.2	5.9	8.2	11.7	13.7	1.08 (0.91–1.27)
Risperidone	261	3.2	5.3	6.7	9.9	13.0	Reference
Ischemic stroke							
Aripiprazole	370	4.3	6.8	10.4	14.3	17.8	1.10 (0.95–1.28)
Risperidone	320	4.1	7.2	9.6	11.7	16.0	Reference
Hemorrhagic stroke							
Aripiprazole	62	0.6	1.2	2.0	2.7	2.7	1.47 (0.99–2.18)
Risperidone	40	0.5	0.7	1.0	1.5	2.8	Reference
Heart failure							
Aripiprazole	807	9.8	16.3	22.1	28.3	33.7	**1.29 (1.16–1.44)**
Risperidone	607	7.8	12.9	17.3	21.7	27.9	Reference
Ventricular arrhythmia							
Aripiprazole	278	3.0	5.2	7.9	11.0	15.0	**1.29 (1.08–1.54)**
Risperidone	205	2.2	4.4	5.9	8.8	12.3	Reference
Sudden cardiac death							
Aripiprazole	501	4.9	9.3	14.1	18.4	22.8	0.95 (0.84–1.07)
Risperidone	502	5.6	10.1	14.1	18.8	23.6	Reference

^a^ Hazard ratio for outcomes among Aripiprazole group compared to Risperidone group subjects (after propensity score matching). 95% CI, 95% confidence interval.

Bold values indicate statistical significance (*p* < 0.05).

In contrast, atherothrombotic events, specifically MI and ischemic stroke did not significantly differ between the two treatment groups. The incidence of MI over 10 years was approximately 13.7% with aripiprazole vs. 13.0% with risperidone (HR 1.08, 95% CI 0.91–1.27; Table 4). Incidence of ischemic stroke was ∼17.8% vs. 16.0% (HR 1.10, 0.95–1.28). The occurrence of hemorrhagic stroke was relatively rare (≤3% of patients in each group by 10 years). Numerically more hemorrhagic strokes occurred with aripiprazole (62 patients, 10-year risk ∼2.7%) than with risperidone (40 patients, ∼2.8% risk), corresponding to HR 1.47 with a wide 95% CI 0.99–2.18. While this suggested a possible trend toward increased hemorrhagic stroke with aripiprazole, it did not reach statistical significance and event counts were low. Finally, mortality outcomes were captured within the composite; we note that sudden cardiac death occurred in similar numbers in each group (501 vs. 502 patients, essentially identical 10-year incidence ∼22.8% vs. 23.6%). The hazard ratio for sudden cardiac death with aripiprazole was 0.95 (0.84–1.07), indicating no significant difference.

Table 5 present the hazard ratios for MACE in various demographic subgroups. The increased risk associated with aripiprazole was consistent across subgroups, with no significant interactions detected. For sex, the HR for MACE with aripiprazole was 1.02 (0.92–1.13) in women and 1.11 (0.99–1.23) in men (interaction *p* = 0.27). Across age categories, HRs were likewise similar: among patients aged <50 years, HR = 1.08 (0.93–1.25); 50–64 years, HR = 1.09 (0.98–1.22); and ≥65 years, HR = 1.11 (0.95–1.30). There was no evidence that the relative effect of aripiprazole differed by age (*p* for interaction = 0.99). Among Asian patients (n = 338), the hazard ratio appeared higher (HR ∼1.37) but the confidence interval was very wide (0.86–2.19) due to the small sample.

**TABLE 5:** Subgroup analyses of major adverse cardiovascular events: Hazard ratios comparing aripiprazole with risperidone.

	No. of event/N		Hazard ratio^a^ (95% CI)
	Aripiprazole	Risperidone	
Sex			
Female	695/2,491	658/2,491	1.02 (0.92–1.13)
Male	717/2,829	652/2,829	1.11 (0.99–1.23)
Age, years			
18–49	365/2,113	317/2,113	1.08 (0.93–1.25)
50–64	637/2,091	583/2,091	1.09 (0.98–1.22)
≥65	311/821	300/821	1.11 (0.95–1.30)
Race			
Black	485/1,894	472/1,894	0.97 (0.85–1.10)
White	728/2,434	656/2,434	1.10 (0.99–1.23)
Asian	39/169	32/169	1.37 (0.86–2.19)

^a^ Hazard ratio for outcomes among Aripiprazole group compared to Risperidone group subjects (after propensity score matching). 95% CI, 95% confidence interval.

## Discussion

In this large real-world study of patients with coexisting type 2 diabetes and schizophrenia, we found that aripiprazole was associated with a modest but significantly higher risk of MACE compared to risperidone. Over up to 10 years of follow-up, aripiprazole-treated patients had about a 10% higher hazard of experiencing a MACE, despite extensive matching on baseline risk factors. The absolute incidence of cardiovascular events was high in both groups, reflecting the compounded risk in this comorbid population; nevertheless, those initiated on aripiprazole experienced slightly worse outcomes over time. This finding was somewhat unanticipated, as we had hypothesized that aripiprazole’s more favorable metabolic profile might translate into equal or lower cardiovascular risk. Instead, our data suggest that aripiprazole did not confer a cardioprotective advantage over risperidone in patients with diabetes and may even be associated with a small detriment in long-term cardiovascular outcomes.

Prior literature comparing antipsychotic-specific cardiovascular outcomes is sparse, especially in patients with established metabolic disease. Our results contrast with those of Citrome et al., who reported that risperidone was linked to higher risks of stroke and heart failure relative to aripiprazole (Citrome et al., 2013). In that analysis, aripiprazole was the reference agent and appeared safer than several SGAs (olanzapine, quetiapine, risperidone) on composite CV outcomes. There are several possible reasons for the discrepancy. First, our study population all had type 2 diabetes, a potent risk factor that may overshadow modest medication differences. It is conceivable that in the setting of diabetes, where baseline atherosclerotic risk is very high, any metabolic advantages of aripiprazole (such as less weight gain or lipid increase) are not enough to meaningfully reduce macrovascular events, especially within the follow-up time frame. Second, differences in study design and outcome definitions matter: Citrome and colleagues relied on insurance claims and focused on acute events like stroke and myocardial infarction. We, using EHR data, included a broader array of outcomes including heart failure and arrhythmias, which emerged as key differentiators in our cohort. Notably, we found a significantly higher incidence of new-onset heart failure in aripiprazole users, whereas Citrome et al. found risperidone had higher heart failure risk. This could reflect differences in patient characteristics (our patients already had diabetes and perhaps end-organ damage) or differences in how heart failure was identified and managed across the populations.

Another relevant comparison is with studies of metabolic outcomes. Aripiprazole is widely considered to have a lower propensity for weight gain and adverse metabolic effects than risperidone (Scigliano and Ronchetti, 2013). Our own baseline data support that notion indirectly: clinicians may have preferentially prescribed aripiprazole to patients who already had obesity or poor glycemic control (since those factors were more prevalent pre-match among aripiprazole users), possibly expecting a more weight-neutral effect. Clinical trials in non-diabetic schizophrenia patients have shown comparable weight gain on aripiprazole vs. risperidone over 1 year, although aripiprazole caused less elevation in triglycerides in one study (Vázquez-Bourgon et al., 2022). A meta-analysis of early-course schizophrenia treatment found aripiprazole more favorable than risperidone in terms of weight gain risk (Kim et al., 2021). These metabolic differences, however, did not translate into fewer cardiovascular events with aripiprazole in our diabetic cohort. This underscores that metabolic risk factors are only part of the equation in determining CV outcomes. Factors unrelated to blood glucose or lipids might be at play, such as direct drug effects on the cardiovascular system or differences in blood pressure and autonomic profiles.

Why might aripiprazole be associated with higher rates of heart failure and arrhythmias? One hypothesis involves pharmacologic differences beyond metabolic effects. Aripiprazole’s mechanism as a D_2_ partial agonist and 5-HT_1A agonist can have activating properties, it is less sedating and can cause akathisia (restlessness) more frequently than risperidone, which is a full D_2_/5-HT_2A antagonist with sedative and hypotensive effects. An increased sympathetic tone due to aripiprazole could contribute to cardiovascular stress (Scigliano and Ronchetti, 2013). Excess sympathetic activation is known to precipitate arrhythmias and promote cardiac remodeling and heart failure (Amerena and Julius, 1995). Patients on aripiprazole in our study may have had higher adrenergic activity on average, potentially increasing the likelihood of tachyarrhythmias or exacerbating latent cardiac dysfunction. In contrast, risperidone’s stronger antagonism at α1-adrenergic receptors causes more orthostatic hypotension and might paradoxically reduce cardiac workload slightly. This could partly explain the heart failure finding, risperidone might modestly lower blood pressure or have mild cardiodepressant effects, which in a failing heart could be beneficial.

The arrhythmia outcome was intriguing. Typically, concern about antipsychotic-induced arrhythmias centers on QT interval prolongation leading to torsade de pointes. Risperidone is known to prolong the QT interval to a moderate degree, whereas aripiprazole has minimal QT prolongation effect. One might therefore expect risperidone to pose higher arrhythmic risk. However, our data showed more ventricular arrhythmias in the aripiprazole group. There are a few interpretations: (1) Not all ventricular arrhythmias are due to long QT; catecholamine-driven ventricular tachycardia or ischemia-related arrhythmias could be more relevant here. Aripiprazole’s tendency to cause akathisia and insomnia might lead to higher catecholamine levels, precipitating arrhythmias in susceptible individuals (Scigliano and Ronchetti, 2013). (2) There may have been residual confounding, for example, if aripiprazole was preferentially given to patients with a history of QT prolongation or who could not tolerate other antipsychotics, those patients might inherently have had a higher arrhythmic risk.

Although aripiprazole is generally regarded as cardiometabolically favorable, emerging case-based evidence suggests it may carry electrophysiologic risks beyond QT prolongation. A 13-year-old girl developed ventricular arrhythmia while receiving aripiprazole in combination with traditional Chinese medicine, likely due to elevated serum drug levels and pharmacodynamic interactions (Shao et al., 2015). Another report described a 43-year-old man who developed new-onset atrial fibrillation during rapid aripiprazole titration, potentially influenced by cardiovascular comorbidities, CYP2D6 polymorphism, and concurrent medications (D’Urso et al., 2018). These cases indicate that aripiprazole may disrupt cardiac conduction through mechanisms unrelated to QT prolongation. Dysregulation of G-protein-coupled receptor signaling has been proposed, potentially altering calcium handling or sympathetic tone, thereby promoting arrhythmia (Ang et al., 2012).

From a clinical perspective, our study suggests that when treating patients with schizophrenia who also have T2DM, choosing aripiprazole over risperidone may not provide a cardiovascular benefit and could be associated with a slightly elevated risk of certain cardiovascular outcomes. This is an important consideration for interdisciplinary care of these patients. It may be prudent for clinicians to monitor cardiovascular health closely regardless of which antipsychotic is used. Specifically, given the heightened incidence of heart failure observed with aripiprazole, clinicians should be vigilant for early signs of cardiac dysfunction (e.g., new edema, dyspnea, or fatigue on exertion) in patients on aripiprazole, especially those with other heart failure risk factors. Baseline and periodic assessment of cardiac function might be considered in high-risk individuals. Similarly, considering the arrhythmia findings, baseline ECG and electrolyte monitoring are reasonable for patients starting any antipsychotic, including aripiprazole, even though it has a low QT effect. Ensuring modifiable arrhythmia risk factors (like hypokalemia or concomitant QT-prolonging drugs) are managed is wise.

For risperidone, while it did not show excess MACEs in this study, it is not necessarily cardioprotective, rather, it performed comparably or slightly better in this diabetic population. Clinicians should continue to mitigate risperidone’s known side effects (such as weight gain, hyperprolactinemia, and sedation) and implement lifestyle and pharmacologic interventions to control diabetes and cardiovascular risk factors. The fact that over half of these patients had a major CV event within 10 years is a stark reminder that aggressive risk factor management is imperative in patients with dual diagnoses. Our findings do not imply that risperidone is “safe,” only that aripiprazole was not safer in this context. Both medications require careful management of the patient’s overall health.

This study has several limitations inherent to its observational design. Despite rigorous propensity matching on many covariates, residual confounding may be present. We balanced groups on diagnoses of major comorbidities and on broad categories of medications and utilization, but we could not account for unmeasured factors such as dietary habits, severity and duration of schizophrenia (e.g., negative symptoms or level of functioning), or physician prescribing biases. For instance, if clinicians tended to prescribe aripiprazole to patients who had more refractory illness or who had already failed other antipsychotics, those patients might have had more cumulative exposure to other medications or longer illness duration that could predispose them to worse outcomes.

Another limitation is that medication exposure was inferred from prescriptions/orders in the EHR. We did not have data on serum drug levels or definite confirmation that patients were taking the medications as prescribed. Non-adherence could blur distinctions between groups. Additionally, this study was unable to account for the specific dosages of antipsychotic medications. The TriNetX network does not provide standardized or complete data on prescribed doses across contributing sites, which limits our ability to assess dose-dependent effects or conduct stratified analyses by dosage levels. As antipsychotic dose may significantly influence both therapeutic efficacy and adverse cardiovascular risk, the lack of dosage adjustment represents a potential unmeasured confounder that should be considered when interpreting our findings. Furthermore, although we employed a 1-year washout period to define new users, it is possible that some patients may have previously used the same or other antipsychotic agents prior to that window. Therefore, our cohort may include individuals re-initiating treatment after a prolonged gap rather than being entirely antipsychotic-naïve, which may introduce exposure misclassification.

Despite limitations, our study has notable strengths. It leveraged a large sample size with substantial follow-up, allowing us to assess relatively rare outcomes like MI and stroke with adequate power. The use of a real-world database increases clinical applicability, as our cohort likely reflects the complexity of patients seen in practice rather than the highly selected patients in randomized trials. We comprehensively adjusted for a wide range of confounders, including granular data on obesity, HbA1c, and medication use, which many database studies lack. Another strength is the examination of a meaningful composite outcome (MACE) in line with what cardiology and endocrinology guidelines consider critical endpoints, and the decomposition of this composite to understand the patterns. By focusing on patients with diabetes, our study directly addresses a gap in evidence for a population at the crossroads of psychiatry and endocrinology, providing insight relevant to both specialties.

The clinical take-home is that in patients with both T2DM and schizophrenia, aripiprazole did not outperform risperidone in terms of cardiovascular safety. The difference observed, a hazard ratio of 1.10, is modest, suggesting that if aripiprazole confers risk, it is not a dramatic increase, but over a large population it could be clinically relevant (e.g., 2.6% absolute risk difference at 10 years in our cohort). This result may reflect a balance of effects: aripiprazole’s slight metabolic advantages being offset by other physiological impacts that increase CV risk. It serves as a reminder that “metabolically friendly” is not the same as “cardio-protective.” All antipsychotics should be prescribed with caution in patients with T2DM, and efforts to mitigate cardiovascular risk should be aggressive regardless of the psychiatric treatment chosen.

Further research is warranted to confirm our findings and clarify mechanisms. Prospective studies or randomized trials specifically in patients with psychiatric illness and metabolic disorders would be ideal but are logistically challenging. Alternatively, large observational studies in other databases (including non-US populations) could validate whether the slight risk increase with aripiprazole is consistent. Biological studies examining the effects of aripiprazole on sympathetic activity, insulin signaling, and cardiac myocyte function could shed light on why heart failure risk might be elevated. It would also be useful to investigate if certain subgroups might do better on one drug, for instance, does baseline heart health modify the effect of antipsychotic choice? And, importantly, studying other SGAs in the context of diabetes (e.g., olanzapine vs. risperidone in diabetics) would help complete the picture of how to tailor antipsychotic selection to medical comorbidity profiles.

In conclusion, our study contributes evidence that when managing patients with both schizophrenia and T2DM, clinicians should not assume aripiprazole is a risk-free choice for the heart. Risperidone appeared at least as safe, if not slightly safer, from a cardiovascular standpoint over long-term follow-up. The absolute cardiovascular risk in this population is high, calling for proactive risk reduction. Ultimately, the integration of psychiatric and medical care is essential to improve outcomes in these patients. By recognizing the nuanced effects of antipsychotic medications on cardiovascular health, we can better stratify risk and personalize treatment—striving to reduce the mortality gap that persists for people living with serious mental illness and metabolic disease.

## Data Availability

This population-based study obtained data from the TrinetX platform (accessible at https://trinetx.com/), for which third-party restrictions apply to the availability of this data. The data were used under license for this study with restrictions that do not allow for data to be redistributed or made publicly available. To gain access to the data, a request can be made to TriNetX (join@trinetx.com), but costs might be incurred, and a data-sharing agreement would be necessary. For inquiries related to the dataset or the study, please contact the corresponding author, Dr. Edy Kornelius, at korn3lius82@gmail.com.
