# Supplementary material for: Comparative cardiovascular outcomes of aripiprazole vs. risperidone in patients with type 2 diabetes and schizophrenia: a retrospective cohort study
Source: Front Pharmacol. 2025 Jul 28;16:1617534. doi: 10.3389/fphar.2025.1617534 (PMC12336492; doi:10.3389/fphar.2025.1617534)
Supplement: Supplementary file 1 [file Table1.docx]

**Supplemental table 1.** Definitions of covariates

| **Co-variates (within 1 year before index date)** | **Code(s)** |
| --- | --- |
| Type 2 Diabetes mellitus | E11 |
| Schizophrenia | F20 |
| Aripiprazole | N05AX12, RxNorm 89013 |
| Risperidone | N05AX08 |
|  |  |
| **Major adverse cardiac events (MACEs)** |  |
| Myocardial infarction | ICD10 = I21−I22 |
| Ischemic stroke | ICD10 = I63, I65, I66, I67.89 |
| Hemorrhagic stroke | ICD10 = I61−I62 |
| Heart failure | ICD10 = I50 |
| Ventricular arrhythmia | ICD10 = I47.0, I47.2, I49.3, I49.0 |
| Sudden cardiac death | ICD9 = I46 |
| Mortality |  |
|  |  |
| **Age at index date** |  |
| **Sex** |  |
| **Ethnicity** |  |
| Not Hispanic or Latino |  |
| Hispanic or Latino |  |
| Unknown Ethnicity |  |
| **Race** |  |
| American Indian or Alaska Native | 1002-5 |
| Asian | 2028-9 |
| Black or African American | 2054-5 |
| Native Hawaiian or Other Pacific Islander | 2076-8 |
| Unknown Race | 2131-1 |
| White | 2106-3 |
| **BMI** | 9083 |
| **Medical utilization** |  |
| Office or Other Outpatient Services | CPT:1013626 |
| Hospital Inpatient Services | CPT:1013659 |
| Emergency Department Services | CPT: 1013711 |
| **Socioeconomic and psychosocial circumstances** | Z55-Z65 |
| Problems related to education and literacy | Z55 |
| Problems related to employment and unemployment | Z56 |
| Occupational exposure to risk factors | Z57 |
| Problems related to housing and economic circumstances | Z59 |
| **Lifestyle** |  |
| Tobacco use | Z72.0 |
| Nicotine dependence | F17 |
| Alcohol related disorders | F10 |
| **Comorbidities** |  |
| Hypertensive diseases | I10-I1A |
| Diabetes mellitus | E08-E13 |
| Disorders of lipoprotein metabolism and other lipidemias | E78 |
| Neoplasms | C00-D49 |
| Chronic kidney disease (CKD) | N18 |
| Ischemic heart diseases | I20-I25 |
| Cerebrovascular diseases | I60-I69 |
| Atrial fibrillation and flutter | I48 |
| Heart failure | I50 |
| Other chronic obstructive pulmonary disease | J44 |
| Asthma | J45 |
| Diseases of liver | K70-K77 |
| Sleep disorders | G47 |
| **Medications** |  |
| Biguanides | A10BA |
| Sulfonylureas | A10BB |
| Alpha glucosidase inhibitors | A10BF |
| Thiazolidinediones | A10BG |
| Dipeptidyl peptidase 4 (DPP-4) inhibitors | A10BH |
| Glucagon-like peptide-1 (GLP-1) analogues | A10BJ |
| Sodium-glucose co-transporter 2 (SGLT2) inhibitors | A10BK |
| HMG CoA reductase inhibitors | C10AA |
| Aspirin | 1191 |
| BETA BLOCKERS/RELATED | CV100 |
| ALPHA BLOCKERS/RELATED | CV150 |
| LOW-CEILING DIURETICS, THIAZIDES | C03A |
| CALCIUM CHANNEL BLOCKERS | CV200 |
| **Laboratory** |  |
| HbA1c, % | 9037 |
| eGFR, mL/min/1.73m^2^ | 8001 |
